# Supplementary material for: N-3 Fatty Acids in Seafood Influence the Association Between the Composite Dietary Antioxidant Index and Depression: A Community-Based Prospective Cohort Study
Source: Antioxidants (Basel). 2024 Nov 18;13(11):1413. doi: 10.3390/antiox13111413 (PMC11591020; doi:10.3390/antiox13111413)
Supplement: Supplementary file 1 [file antioxidants-13-01413-s001.zip › antioxidants-3230235-supplementary.pdf]

Supplementary Table S1. Classification of food items.

**Food groups**	**Food items**
Grains	White rice, rice with beans, rice with other cereals, half & half rice with beans, half & half rice with other cereals, ramyon, wheat noodles, Chajangmyon/Jambbong, cold noodles/buckwheat noodles, dumpling/dumpling with soup, white rice cake/rice cake with soup, other rice cakes, corn flakes, loaf bread/sandwich/toast, bread with small red beans, other breads, cakes/chocopie, pizza/hamburger, parched cereal powder, starch jelly
Potatoes	Potatoes, sweet potatoes, starch vermicelli
Legumes	Beans/beans cooked in soy sauce, soup and stew with soybean paste/soybean paste, tofu, soybean milk
Nuts and seeds	Peanuts/almonds/pine nuts
Fruits	Strawberry, oriental melon/melon, watermelon, peach/plum, banana, persimmon/dried persimmon, tangerine, pear/pear juice, apple/apple juice, orange/orange juice, grape/grape juice, tomato/cherry tomato/tomato juice
Vegetables	Korean cabbages/Korean cabbage soup, spinach, lettuce, perilla leaf, vegetables wrap/vegetable salad, other green vegetables, deoduck/doraji, bean sprouts, bracken/sweet potato stalk/stem of taro, pepper leaves/chamnamul/asterscaber, crown daisy/chives/water dropwort, cucumber, carrot/carrot juice, onion, green pepper, zucchini, pumpkin/kabocha squash/pumpkin juice
Mushrooms	Oyster mushroom, other mushrooms
Meats	Pork belly, roasted pork, braised pork, processed meat(ham/sausage), edible viscera, steak/roasted beef, dog meat, fried chicken/chicken stew, beef soup, beef soup with vegetables
Eggs	Eggs
Fish and shellfish	Sushi, mackerel/pacific saury/Spanish mackerel, hair tail, eel, yellow croaker/sea bream/flat fish, Alaska pollack, squid/dried squid/octopus, dried anchovy, canned tuna, salt-fermented fish, clam/whelk, oyster, crab, shrimp, fish paste/crab flavored
Seaweeds	Dried laver, kelp/sea mustard
Milk and dairy products	Milk, yogurt, ice cream, cheese
Oils and sugars	Jam/honey/butter/margarine, coffee cream
